# TI-YOLO: A Lightweight and Efficient Anatomical Structure Detection Model for Tracheal Intubation

**DOI:** 10.3390/bioengineering13040451

**Published:** 2026-04-13

**Authors:** Yu Tian, Congliang Yang, Lingfeng Sang, Cicao Ping, Lili Feng, Weixiong Chen, Hongbo Wang, Wenxian Li, Yuan Han

**Affiliations:** 1 Department of Anesthesiology, Eye & ENT Hospital of Fudan University, No. 83 Fenyang Road, Xuhui District, Shanghai 200031, Chinayclone123@gmail.com (C.Y.); fenglili@eentanesthesia.com (L.F.); feynmanx@outlook.com (W.C.); 2 Ningbo Key Laboratory of Aging Health Equipment and Service Technology, Ningbo Polytechnic, Ningbo 315800, China; sanglingfeng@163.com (L.S.); pingcicao@gmail.com (C.P.); 3 School of Health Science and Engineering, University of Shanghai for Science and Technology, No. 516 Jungong Road, Yangpu District, Shanghai 200093, China; 4 College of Intelligent Robotics and Advanced Manufacturing, Fudan University, No. 220 Handan Road, Yangpu District, Shanghai 200433, China; wanghongbo@fudan.edu.cn

**Keywords:** tracheal intubation, feature fusion, attention mechanism, lightweight

## Abstract

Accurate and rapid detection of anatomical structures, such as the glottis, is critical during tracheal intubation (TI) to ensure patient safety and procedural success. However, it remains a challenge due to the limited field of view and computational resources of video laryngoscopy, especially for difficult airway situations. Existing deep learning (DL) models struggle to balance high accuracy and real-time clinical deployment. To address these issues, we propose TI-YOLO (TI-You Only Look Once), a lightweight and efficient object detection model built upon the YOLOv11 architecture. TI-YOLO introduces the Bidirectional Feature Pyramid Network (BiFPN) module for multi-scale feature fusion, effectively enhancing the ability to detect anatomical structures of different sizes. TI-YOLO integrates the Deformable Attention Transformer (DAT) module to enhance the perception of crucial regions, improving detection accuracy and robustness. To further reduce the consumption of computational resources while maintaining efficiency, TI-YOLO is optimized by reconstructing the backbone based on MobileNetV4. Furthermore, TI-YOLO employs the Slide Weight Function (SWF) as a loss function during model training to mitigate the class imbalance within the dataset. One self-built dataset is used to validate the effectiveness of TI-YOLO. Compared to the original YOLOv11, TI-YOLO achieves mean Average Precision at IoU 0.50 (mAP50) scores of 0.902, with improvements of 3.8%. Meanwhile, TI-YOLO balances detection accuracy and computational efficiency with a 10.5% reduction in floating-point operations (FLOPs) and a 28.9% reduction in parameters, and the model weight is only 4.6 MB. Additionally, to evaluate TI-YOLO real-time inference capability, we quantize and deploy it on a low-cost embedded OrangePi 5 platform. The inference speed reaches over 50 frames per second (FPS), meeting real-time clinical requirements.

## 1. Introduction

Tracheal intubation (TI) is a fundamental and high-risk procedure in surgical anesthesia and critical care, playing a pivotal role in securing airway patency and maintaining adequate ventilation [1]. The safe window for successful intubation is typically limited to approximately three minutes, and failure to accurately identify airway anatomy may lead to severe complications, including hypoxemia, airway trauma, or even fatal outcomes [2]. Consequently, improving the accuracy and efficiency of anatomical structure recognition during TI is of critical clinical importance.

Video laryngoscopy has become the mainstream tool for modern airway management due to its superior visualization compared with direct laryngoscopy. By providing real-time video feedback, it allows clinicians to observe anatomical landmarks such as the glottis, epiglottis, and vocal cords, thereby improving intubation success rates [3]. However, in emergency or difficult airway scenarios, rapid and accurate identification of these structures remains challenging, particularly for junior anesthesiologists and non-anesthesiologists who perform intubation infrequently [4]. Limited field of view, anatomical variability, occlusion by soft tissues, and motion-induced blur further exacerbate the difficulty of reliable visual interpretation.

Recent advances in artificial intelligence (AI), especially deep learning (DL), have significantly promoted progress in medical image analysis and endoscopic assistance systems. Convolutional neural networks (CNNs) and transformer-based models have demonstrated strong capabilities in gastrointestinal endoscopy, bronchoscopy, and cystoscopy for tasks such as lesion detection, segmentation, depth estimation, and image enhancement [5,6,7]. In the context of airway management, AI has been explored across the entire clinical workflow, including difficult airway prediction, real-time anatomical recognition, and operator training [8,9,10]. For instance, CNN-based and hybrid YOLO–U-Net models have been proposed for detecting the glottis and epiglottis, achieving high segmentation accuracy under controlled conditions [11,12,13,14].

Despite these encouraging advances, applying AI-assisted algorithms to video laryngoscopy presents unique challenges. Compared with other endoscopic modalities, laryngoscopic images are characterized by a narrow field of view, substantial inter-patient anatomical variability, and subtle texture differences between target structures and surrounding tissues. Rapid camera motion and scale variation during intubation further degrade image quality and increase background confusion. Meanwhile, clinical deployment imposes strict real-time and low-power constraints: embedded video laryngoscope platforms typically possess limited computational resources, making it difficult to deploy large-scale deep neural networks without compromising inference speed or device endurance.

To address these challenges, recent research in object detection has focused on enhancing feature representation and computational efficiency. Multi-scale feature fusion mechanisms, such as Feature Pyramid Networks (FPNs), Path Aggregation Networks (PANs), and Bidirectional Feature Pyramid Networks (BiFPNs), have been widely adopted to improve the detection of objects with varying sizes by aggregating hierarchical features [15,16,17,18]. In parallel, attention mechanisms—including deformable convolution and deformable attention transformers—have demonstrated effectiveness in focusing on spatially relevant regions and improving robustness under occlusion and geometric variation [19,20,21]. Furthermore, lightweight network architectures such as MobileNet and model compression techniques based on quantization-aware training (QAT) have been developed to enable efficient deployment on resource-constrained devices while minimizing performance degradation [22,23,24].

Motivated by these observations, this study aims to design a lightweight yet accurate object detection framework tailored for real-time anatomical structure recognition during tracheal intubation. In this paper, we propose TI-YOLO (TI-You Only Look Once), an efficient detector built upon the YOLOv11 architecture. TI-YOLO integrates a BiFPN to enhance multi-scale feature fusion and incorporates a Deformable Attention Transformer (DAT) module to strengthen the perception of critical anatomical regions. To further reduce computational overhead, the backbone network is reconstructed based on MobileNetV4, and a Slide Weight Function (SWF) loss is adopted to alleviate class imbalance during training. Additionally, quantization-aware training is employed to facilitate deployment on low-cost embedded platforms.

The main contributions of this work can be summarized as follows:

- We propose TI-YOLO, a lightweight and efficient detector of anatomical structure for tracheal intubation. TI-YOLO is implemented through improvements based on the YOLOv11 architecture. By integrating the DAT and BiFPN modules, the feature extraction ability for structures of different sizes is significantly enhanced, while reducing the impact of background confusion. To reduce model complexity, the backbone of TI-YOLO is reconstructed using MobileNetV4.
- We integrate SWF as the loss function and introduce the QAT method to achieve high real-time performance for deployment on resource-constrained hardware devices.
- We conducted experiments on one self-built dataset. The experimental results demonstrate that TI-YOLO has advanced performance in detection tasks and outperforms several benchmark models across multiple metrics.

## 2. Materials and Methods

### 2.1. Dataset Description

**Figure 1:**
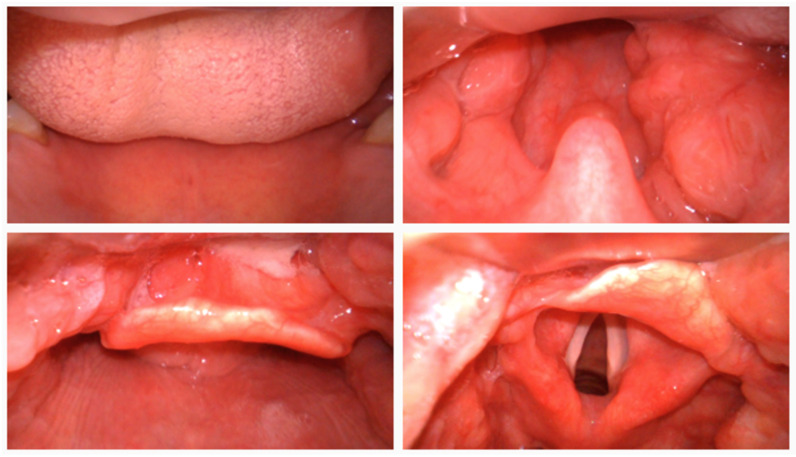
Sample images from the self-constructed laryngoscopic dataset.

To comprehensively evaluate the effectiveness of the proposed TI-YOLO model, a self-constructed laryngoscopic dataset was developed to serve as the primary benchmark for performance assessment under realistic tracheal intubation scenarios, sample raw images are shown in Figure 1. The dataset was acquired using a video laryngoscope in a simulated tracheal intubation environment. All data collection procedures were conducted under controlled conditions to ensure consistent image quality while preserving clinically relevant variability. Each image frame was manually annotated with bounding boxes corresponding to key anatomical structures involved in the intubation process.

The dataset comprises a total of 4140 images from 1300 samples, which were partitioned into 3726 training images and 414 validation images. This retrospective study was registered in the Chinese Clinical Trial Registry (ChiCTR, Registration No. ChiCTR2500107435). Anatomical categories were defined according to their clinical relevance to the intubation procedure, including teeth, tongue, uvula, epiglottis, glottis, Cormack–Lehane (CL) grades, carina, and esophagus. Notably, the glottis category was further subdivided based on the Cormack–Lehane grading system to more effectively characterize variations in glottic exposure associated with intubation difficulty.

In addition, a five-fold cross-validation scheme was employed on the 3726-image training subset to minimize split-induced bias and detect overfitting. The subset was randomly stratified into five disjoint folds; in each of the five rounds, four folds trained the model while the fifth served for internal validation, guaranteeing full coverage. Concurrently, a separate 414-image dataset was maintained as a held-out test set for unbiased final reporting.

### 2.2. Overall Architecture of TI-YOLO

**Figure 2:**
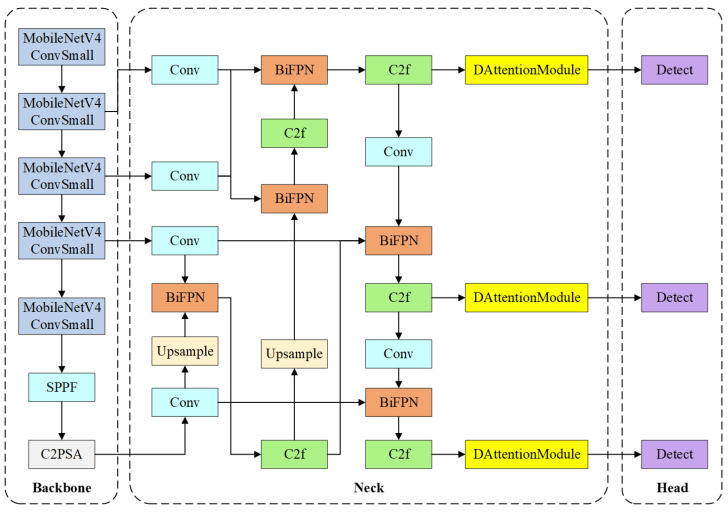
Overall Architecture of Proposed TI-YOLO.

YOLOv11 is selected as the baseline framework due to its lower parameter count compared with the latest YOLOv8 while maintaining competitive detection accuracy in object detection tasks. The overall architecture of TI-YOLO is illustrated in Figure 2. TI-YOLO follows the standard object detection paradigm and consists of three main components: a lightweight backbone for feature extraction, a multi-scale neck for feature fusion, and a detection head for anatomical structure localization and classification. The design of TI-YOLO is guided by the dual objectives of achieving high detection accuracy and preserving real-time performance under constrained computational resources.

In the backbone stage, the original YOLOv11 backbone is reconstructed using MobileNetV4 blocks to substantially reduce model complexity and computational cost while preserving adequate feature representation capability. The backbone progressively extracts hierarchical feature maps at multiple resolutions, which are subsequently fed into the neck module for further processing. In addition, to further enhance the representational capacity of the backbone output features, a Spatial Pyramid Pooling-Fast (SPPF) module and a C2PSA module are introduced at the end of the backbone. The SPPF module enlarges the receptive field and aggregates multi-scale contextual information through a series of lightweight pooling operations, thereby improving the ability to capture anatomical structures with varying sizes. The C2PSA module further refines the high-level features by incorporating an attention mechanism into the lightweight C2 structure, which helps emphasize clinically important regions and suppress background interference.

The neck of TI-YOLO adopts a BiFPN to achieve efficient multi-scale feature fusion. By enabling both top-down and bottom-up information flow with learnable fusion weights, BiFPN enhances the representation of anatomical structures with diverse spatial scales, such as the glottis, epiglottis, and tongue. In addition, C2f modules are employed to further refine the fused features while maintaining computational efficiency.

In the head stage, DAT modules are integrated before the detection layers. This module can adaptively focus on more informative spatial positions and strengthen responses to critical anatomical regions such as the glottis and epiglottis. In this way, the DAT module functions as a feature refinement component for the neck outputs, improving detection accuracy and robustness with limited additional computational cost. The detection heads then predict bounding box coordinates and category labels for each anatomical structure across multiple scales. Importantly, the incorporation of the BiFPN and DAT modules does not modify the fundamental detection head design of YOLOv11, thereby ensuring compatibility with lightweight deployment and real-time inference.

### 2.3. Lightweight Backbone Reconstruction

To enable efficient deployment under constrained computational resources, TI-YOLO adopts a backbone architecture reconstructed using MobileNetV4. Conventional YOLO backbones are typically built upon deep and computationally intensive convolutional structures to maximize feature representation capacity. Although effective in high-performance computing environments, such architectures introduce considerable parameter redundancy and floating-point computational overhead, which significantly hinders their deployment on embedded video laryngoscope platforms.

MobileNetV4 is designed according to efficiency-oriented principles that emphasize practical performance under hardware constraints. By employing a compact network architecture and optimized convolutional operations, MobileNetV4 achieves competitive feature extraction capability while substantially reducing unnecessary computations. These characteristics make it particularly suitable for real-time inference on low-power embedded devices.

The efficiency of the reconstructed backbone primarily stems from the adoption of lightweight convolutional structures that decouple spatial filtering from channel-wise feature aggregation. Compared with conventional convolutions, this design strategy results in a substantial reduction in both parameter count and floating-point operations (FLOPs), thereby enabling more efficient utilization of computational resources. Despite the reduced model complexity, the backbone retains sufficient representational capacity to support accurate object detection.

By integrating MobileNetV4 into the backbone of TI-YOLO, the proposed model significantly decreases the overall computational burden and model size while still producing stable and discriminative multi-scale feature representations. These features provide reliable inputs for subsequent components, including the BiFPN-based feature fusion module and the DAT module. Consequently, TI-YOLO achieves an effective trade-off between detection accuracy and computational efficiency, enabling real-time anatomical structure recognition on embedded platforms without sacrificing performance.

### 2.4. Multi-Scale Feature Fusion

Accurate detection of anatomical structures during tracheal intubation requires effective integration of multi-scale features, as target objects exhibit substantial variations in size, shape, and visual appearance under different imaging conditions. To address this challenge, TI-YOLO adopts a BiFPN as the neck architecture to improve the efficiency of multi-scale feature fusion.

Conventional Feature Pyramid Network (FPN) architectures primarily rely on a top-down pathway to propagate high-level semantic information to lower-resolution feature maps. Although effective, this strategy provides limited feedback from low-level spatial features to higher layers. Subsequent extensions, such as the Path Aggregation Network (PAN), introduce an additional bottom-up pathway to mitigate this limitation. However, fixed fusion paths and uniform feature weighting may still result in suboptimal information utilization and increased computational overhead.

BiFPN improves upon these architectures by introducing bidirectional information flow and learnable feature fusion weights, enabling more flexible and efficient aggregation of multi-scale representations. Specifically, BiFPN constructs both top-down and bottom-up pathways while eliminating redundant nodes, thereby simplifying the fusion topology and reducing computational cost. The incorporation of learnable weights allows the network to adaptively emphasize more informative feature levels during training, which is particularly advantageous for detecting anatomical structures with large variations in scale.

More specifically, let $\left\{F_{3},F_{4},F_{5}\right\}$ denote the multi-scale feature maps produced by the backbone, corresponding to spatial resolutions from high to low. In the top-down pathway, deep features contain strong semantic information but have coarse spatial resolution; therefore, they are first upsampled and fused with shallower high-resolution features so that fine localization cues can be complemented by richer semantic context. In the bottom-up pathway, the refined high-resolution features are further downsampled and propagated back to deeper layers, allowing precise boundary and positional information to reinforce low-resolution representations. At each fusion node, features from different resolutions are first aligned to the same scale and then aggregated using normalized learnable weights:

(1) $${\hat{F}}_{l}=\frac{\sum_{i}w_{i}\cdot \mathrm{Resize}\left(F_{i}\right)}{\varepsilon +\sum_{i}w_{i}},$$

where $w_{i}$ denotes the learnable contribution weight of the *i*-th input feature, and Resize(·) represents upsampling or downsampling for spatial alignment, and ε is a small positive constant added to the denominator with a value of $10^{-4}$, which is used to avoid division by zero and to improve numerical stability when the sum of fusion weights is very small. In this way, TI-YOLO can simultaneously preserve fine-grained details required for small or partially occluded structures and maintain high-level semantic context for larger anatomical regions, thereby improving detection accuracy and localization robustness across different target scales.

In TI-YOLO, BiFPN modules are stacked and integrated with lightweight convolutional blocks to further enhance feature interaction while preserving computational efficiency. High-resolution feature maps retain fine-grained spatial details that are critical for small or partially occluded structures, whereas low-resolution feature maps provide robust semantic context for larger anatomical regions. Through bidirectional fusion, these complementary characteristics are effectively combined, resulting in more discriminative and scale-aware feature representations.

Overall, the integration of BiFPN enables TI-YOLO to achieve robust multi-scale feature fusion with minimal additional computational overhead. This design improves detection accuracy and stability under challenging laryngoscopic imaging conditions, while remaining compatible with lightweight deployment requirements for real-time clinical applications.

### 2.5. Deformable Attention Transformer Module

**Figure 3:**
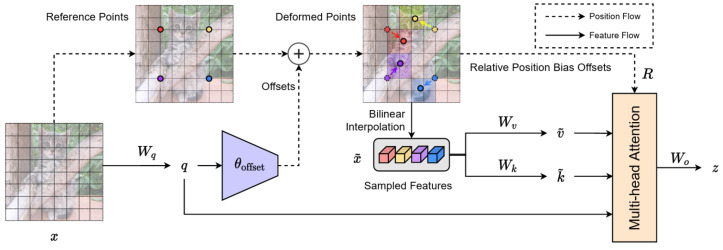
An illustration of information flow of deformable attention.

Laryngoscopic images are characterized by complex anatomical structures, partial occlusion, and strong visual similarity between target regions and surrounding tissues. Conventional self-attention mechanisms in Vision Transformers attend uniformly to all spatial locations, resulting in high computational cost and potentially diluting attention on clinically relevant regions. To overcome these limitations, TI-YOLO incorporates a DAT module to enable sparse and adaptive attention guided by informative spatial regions, which is shown in Figure 3.

Unlike standard attention, which computes interactions between all query and key tokens, deformable attention restricts the attention operation to a small set of dynamically learned sampling points. These sampling points are generated in a data-dependent manner, allowing the model to focus on anatomically critical areas, such as the glottic opening, while suppressing responses from irrelevant background regions.

Given an input feature map $x\in R^{H\times W\times C}$, query embeddings are obtained as:

(2) $$q=xW_{q},$$

where $W_{q}$ denotes the projection matrix. A lightweight offset network $\theta_{\mathrm{offset}}\left(\cdot \right)$ predicts spatial offsets Δp from the query features:

(3) $$\Delta p=s\cdot \tanh\left(\theta_{\mathrm{offset}}\left(q\right)\right),$$

where *s* is a predefined scaling factor to stabilize training and prevent excessive spatial displacement. Based on the reference grid points *p*, the deformed sampling locations are computed as p+Δp. Feature values at these locations are obtained through bilinear interpolation:

(4) $$\tilde{x}=\phi \left(x;p+\Delta p\right),$$

where ϕ(·) denotes the differentiable sampling function. The sampled features are then projected to keys and values:

(5) $$\tilde{k}=\tilde{x}W_{k},\;\tilde{v}=\tilde{x}W_{v}.$$

Multi-head attention is subsequently performed between the queries and the deformed keys:

(6) $$z^{\left(m\right)}=\mathrm{softmax}\left(\frac{q^{\left(m\right)}{\tilde{k}}^{(m)T}}{\sqrt{d}}+R\right){\tilde{v}}^{\left(m\right)},$$

where *m* denotes the attention head index, *d* is the feature dimension, and *R* represents relative positional encoding derived from the deformed sampling locations. Outputs from all heads are concatenated and linearly projected to obtain the final refined feature representation.

In TI-YOLO, the DAT module is inserted before each detection head to refine the multi-scale feature maps generated by the BiFPN neck. By adaptively attending to a sparse set of informative spatial locations, the DAT module enhances spatial awareness and improves robustness against occlusion, scale variation, and motion artifacts commonly encountered during tracheal intubation. Importantly, this attention mechanism introduces only minimal additional computational overhead, thereby preserving the real-time inference capability of the overall system.

### 2.6. Loss Function and Optimization Strategy

The distribution of anatomical structures in tracheal intubation images exhibits pronounced class imbalance, where easily recognizable regions dominate the dataset, whereas clinically critical structures, such as the glottis, occur far less frequently. This imbalance causes conventional loss functions to be biased toward easy samples, thereby limiting the model’s ability to learn discriminative representations for hard samples.

To address this issue, TI-YOLO incorporates the SWF as an adaptive sample-weighting strategy during training. SWF dynamically assigns higher weights to hard samples while suppressing the contribution of easy samples, enabling the network to focus more on ambiguous and difficult predictions without introducing excessive hyperparameters.

Let *x* denote the Intersection-over-Union (IoU) between the predicted bounding box and the corresponding ground truth. The average IoU value μ over all positive samples in each batch is used as an adaptive threshold to distinguish easy and hard samples. The Slide Weight Function is defined as:

(7) $$f\left(x\right)=\left\{\begin{array}{cc} 1, & x\leq \mu -\delta \\ e^{1-\mu }, & \mu -\delta <x<\mu \\ e^{1-x}, & x\geq \mu \end{array}\right.$$

where δ is a small constant controlling the smooth transition region around the threshold. Samples near the boundary of μ are assigned larger weights, encouraging the model to emphasize hard and ambiguous cases during optimization. The overall loss function of TI-YOLO is formulated as a weighted combination of classification loss, bounding box regression loss, and objectness loss:

(8) $$L=\sum_{i}f\left(x_{i}\right)\left(L_{cls}^{\left(i\right)}+\lambda_{reg}L_{reg}^{\left(i\right)}+\lambda_{obj}L_{obj}^{\left(i\right)}\right),$$

where $f(x_{i})$ denotes the SWF weight of the *i*-th sample, and $\lambda_{reg}$ and $\lambda_{obj}$ are balancing coefficients for regression and objectness terms, respectively.

**Figure 4:**
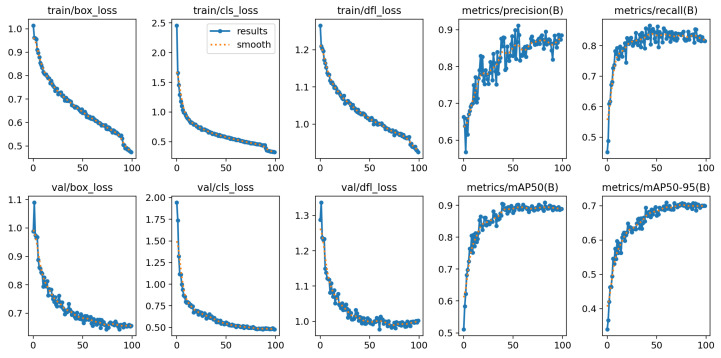
Training Loss and Metric Curves over epochs.

In addition to loss reweighting, the optimization strategy is employed to facilitate efficient deployment on embedded platforms. Specifically, we adopt QAT [25] to simulate low-precision arithmetic during training. In our implementation, both the convolutional weights and activations are quantized to 8-bit integers (INT8), while the first and last layers are kept in floating-point precision to mitigate potential accuracy loss. During QAT, fake-quantization operators are inserted into the network to emulate quantization and dequantization effects in the forward pass, enabling the model parameters to adapt to quantization noise. This strategy effectively minimizes performance degradation after deployment and improves real-time inference efficiency on embedded devices, with the training loss and metric curves over epochs shown in Figure 4.

## 3. Experimental Results

### 3.1. Experimental Setup

Both the proposed TI-YOLO and all comparative baselines were implemented within the PyTorch framework. To ensure experimental reproducibility, the software environment was configured with Python 3.9, CUDA 12.4, PyTorch 2.4.0, OpenCV-Python 4.9.0, and ONNX 1.17.0. Training was conducted on a workstation equipped with two NVIDIA GeForce RTX 4090 GPUs. During training, the Adam optimizer was adopted with an initial learning rate of $1\times 10^{-3}$, and the batch size was set to 32. To evaluate real-time inference performance in practical deployment scenarios, the trained model was further tested on an embedded OrangePi 5 platform.

### 3.2. Evaluation Metrics

To comprehensively evaluate both detection accuracy and computational efficiency of the proposed TI-YOLO model, several standard metrics were employed, including Precision, Recall, Average Precision (AP), Mean Average Precision (mAP), model complexity in terms of FLOPs and parameter count, as well as inference speed measured in frames per second (FPS).

Precision (P): Precision reflects the proportion of correctly predicted bounding boxes among all predicted boxes, indicating the reliability of model predictions. It is computed as:

(9) $$Precision=\frac{TP}{TP+FP},$$

where TP and FP denote the numbers of true positives and false positives, respectively.

Recall (R): Recall evaluates the ability of the model to detect all relevant ground-truth objects and is particularly important for clinical applications where missed detections may lead to serious consequences. It is defined as:

(10) $$Recall=\frac{TP}{TP+FN},$$

where FN represents the number of false negatives.

Average Precision (AP): AP summarizes the precision–recall trade-off for a specific class by computing the area under the Precision–Recall (P–R) curve:

(11) $$AP=\int_{0}^{1}Precision\left(r\right)\;dr,$$

where *r* denotes the recall value.

Mean Average Precision (mAP): mAP provides an overall measure of detection performance by averaging AP across all classes:

(12) $$mAP=\frac{1}{M}\sum_{i=1}^{M}AP_{i},$$

where *M* denotes the total number of anatomical categories. To further clarify, we adopt the widely used variants of mean Average Precision (mAP) as follows:

(13) $$\begin{array}{cc} {\mathrm{mAP}}_{50} & =\frac{1}{M}\sum_{i=1}^{M}AP_{i}\left(\mathrm{IoU}=0.5\right), \end{array}$$

(14) $$\begin{array}{cc} {\mathrm{mAP}}_{50:95} & =\frac{1}{M}\sum_{i=1}^{M}\frac{1}{10}\sum_{t=0.5}^{0.95}AP_{i}\left(\mathrm{IoU}=t\right), \end{array}$$

Here, $mAP_{50}$ refers to the mean Average Precision at a fixed Intersection-over-Union (IoU) threshold of 0.5, providing an intuitive measure of detection performance. In contrast, $mAP_{50:95}$ averages AP across multiple IoU thresholds from 0.5 to 0.95 (in steps of 0.05), offering a stricter evaluation of localization stability and bounding-box regression quality. Both metrics are derived from the per-class AP values defined in Equation (12).

Floating Point Operations (FLOPs): FLOPs represent the number of floating-point operations required to process a single input image. Inference Speed: Measured by Frames Per Second (FPS), indicating how many images can be processed per second.

Among the above metrics, ${\mathrm{mAP}}_{50}$ is used as the primary indicator of overall detection performance in this study, because it provides an intuitive evaluation of detection accuracy under a commonly used IoU threshold. In contrast, ${\mathrm{mAP}}_{50:95}$, which averages performance across multiple IoU thresholds, offers a stricter assessment of localization stability and bounding-box regression quality. Meanwhile, Precision and Recall are reported to characterize false-positive and false-negative behavior, respectively, which is particularly important in tracheal intubation assistance, where false alarms may interfere with anatomical interpretation and missed detections may lead to failure in recognizing critical landmarks. In addition to detection accuracy, FLOPs, parameter count, model size, and FPS are jointly considered to evaluate computational complexity and real-time deployment capability, thereby providing a more comprehensive assessment of the practical value of the proposed method in embedded clinical scenarios.

### 3.3. Model Detection Performance

**Figure 5:**
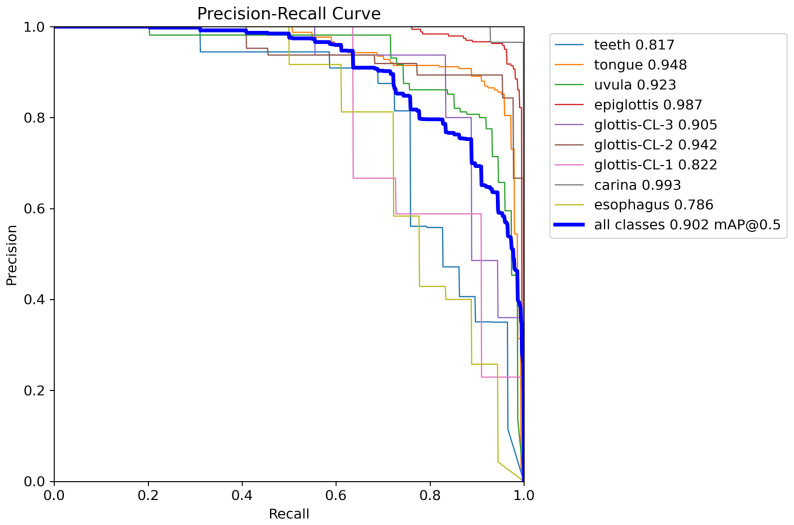
An illustration of Precision–Recall (P-R) curves of TI-YOLO.

The detection performance of different models on the laryngoscopic dataset is summarized in Table 1, and the corresponding Precision–Recall (P-R) curves of TI-YOLO are illustrated in Figure 5. During model development, five-fold cross-validation was conducted on the training subset to improve the robustness of model selection, whereas the final quantitative results reported in Table 1 were obtained on the independent validation set. The proposed TI-YOLO achieves the best overall detection performance and outperforms the baseline models on most anatomical categories.

As reported in Table 1, TI-YOLO achieves an overall ${\mathrm{mAP}}_{50}$ of 0.902 and ${\mathrm{mAP}}_{50:95}$ of 0.447, representing substantial improvements over YOLOv11n (0.703 and 0.397, respectively). These results demonstrate the effectiveness of the proposed architecture in enhancing detection accuracy while preserving a lightweight design. Notable performance gains are observed for clinically critical anatomical structures.

Specifically, TI-YOLO attains an AP of 0.987 for the epiglottis, 0.942 for glottis-CL-2, and 0.905 for glottis-CL-3, which are essential landmarks for guiding successful tracheal intubation. In addition, the model achieves an AP of 0.993 for the carina class, indicating strong robustness in detecting deep airway anatomical structures.

For smaller and more challenging targets, such as glottis-CL-1 and esophagus, TI-YOLO also demonstrates superior performance, with AP values of 0.822 and 0.786, respectively. These improvements suggest that the proposed model effectively captures fine-grained features under conditions of scale variation and partial occlusion.

The P-R curves further validate the stability and reliability of TI-YOLO. Most anatomical categories maintain high precision across a wide range of recall levels, and the aggregated curve yields an area corresponding to an ${\mathrm{mAP}}_{50}$ of 0.902. In particular, the curves for the epiglottis and carina exhibit consistently high precision even at high recall values, indicating strong discriminative capability for key anatomical landmarks.

Overall, these results demonstrate that TI-YOLO achieves a favorable accuracy, making it well-suited for anatomical structure recognition during tracheal intubation.

### 3.4. Ablation Experimental Result

To investigate the contribution of each proposed component in TI-YOLO, ablation experiments were conducted by progressively incorporating the BiFPN module, the DAT module, and the SWF into the baseline model. The results are summarized in Table 2.

The baseline model without any additional modules achieves an ${\mathrm{mAP}}_{50}$ of 0.703. When only the BiFPN module is introduced, the ${\mathrm{mAP}}_{50}$ increases to 0.741, demonstrating the effectiveness of multi-scale feature fusion in improving anatomical structure detection. Similarly, incorporating the DAT module alone raises the ${\mathrm{mAP}}_{50}$ to 0.752, indicating that deformable attention enhances the localization of discriminative regions. The introduction of SWF alone further improves the ${\mathrm{mAP}}_{50}$ to 0.758 by mitigating the adverse effects of class imbalance.

When combining two modules, more pronounced performance gains are observed. The BiFPN + DAT configuration achieves an ${\mathrm{mAP}}_{50}$ of 0.781, whereas the BiFPN + SWF and DAT + SWF combinations reach 0.821 and 0.845, respectively. These results suggest that the proposed modules are complementary and jointly contribute to enhanced detection performance.

The complete TI-YOLO model integrating BiFPN, DAT, and SWF achieves the best overall performance, with a Precision of 0.929, a Recall of 0.855, an ${\mathrm{mAP}}_{50}$ of 0.902, and an ${\mathrm{mAP}}_{50:95}$ of 0.447. The consistent improvements across all evaluation metrics confirm that each component plays a positive and complementary role within the proposed framework.

### 3.5. Embedded Deployment Performance

**Figure 6:**
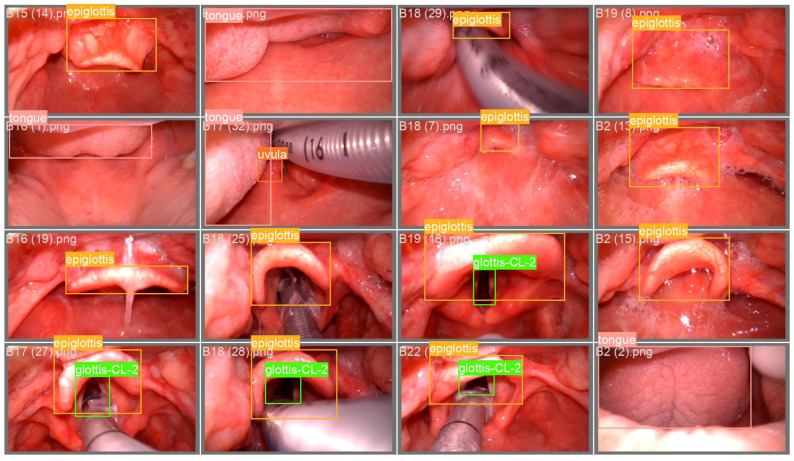
Recognition results of the model deployed on OrangePi.

To evaluate the real-time inference capability of the proposed TI-YOLO model in practical clinical environments, deployment experiments were conducted on a low-cost embedded platform, namely the OrangePi 5. A quantitative comparison of model complexity and deployment performance between YOLOv11n and TI-YOLO is presented in Table 3. Compared with YOLOv11n, TI-YOLO achieves a reduction of 10.8% in GFLOPs and 30.8% in the number of parameters, while the model size is compressed from 5.4 MB to 4.6 MB. This reduction in model complexity improves the feasibility of deployment on resource-constrained embedded devices. In addition, the comparison between the non-quantized and quantized TI-YOLO models further demonstrates the benefit of QAT for embedded deployment. After QAT-based conversion from FP32 to INT8, the number of parameters remains unchanged at 1.8 M, while the model size is further reduced from 4.6 MB to 2.9 MB, and the inference speed on the OrangePi 5 platform increases from 38.6 FPS to over 50 FPS. These results indicate that QAT effectively improves deployment efficiency and real-time performance on embedded hardware. Overall, the embedded deployment experiments validate the practicality of TI-YOLO for real-world clinical applications, demonstrating that the proposed lightweight design achieves a favorable balance between computational efficiency and deployment speed on embedded platforms. Representative recognition examples obtained on the OrangePi 5 platform are shown in Figure 6, demonstrating that the deployed TI-YOLO model can accurately localize key anatomical structures under practical embedded inference conditions.

## 4. Limitations and Future Work

Although the proposed TI-YOLO model demonstrates promising performance in detecting anatomical structures during tracheal intubation, several limitations should be acknowledged.

First, the experimental evaluation is conducted on a self-constructed laryngoscopic dataset, the dataset size remains relatively limited and does not fully cover the diversity of real-world clinical conditions. Future work will focus on collecting larger-scale and multi-center clinical datasets to further validate the generalization ability of the proposed model.

Second, although the proposed model achieves real-time performance on an embedded platform, further optimization is still possible. Advanced model compression techniques, including neural architecture search, structured pruning, and mixed-precision quantization, could be explored to further reduce computational cost and power consumption while maintaining detection accuracy.

Third, the current system is evaluated as an assistive perception module and has not yet been integrated into a complete clinical decision-support pipeline. Future work will aim to combine the proposed detection model with clinical guidance strategies, such as automatic intubation path suggestion and risk warning mechanisms, to provide more comprehensive assistance for anesthesiologists and emergency physicians during airway management.

In addition, because the present study is conducted on a self-constructed laryngoscopic dataset and a unified public benchmark for anatomical structure detection during tracheal intubation is still lacking, it remains difficult to perform a strictly consistent numerical comparison with previously published state-of-the-art methods. Future work will therefore focus on constructing larger-scale multi-center datasets and promoting more standardized benchmarks to improve cross-study comparability.

In summary, future research will focus on expanding clinical datasets, optimizing embedded deployment, and developing an integrated intelligent assistance system for tracheal intubation. These efforts will promote the translation of the proposed approach from laboratory research to real-world clinical applications.

## 5. Conclusions

In this study, a lightweight and efficient object detection framework, termed TI-YOLO, was proposed for automatic recognition of anatomical structures during tracheal intubation. By integrating three innovative modules and adopting an improved loss function, TI-YOLO significantly outperforms several state-of-the-art lightweight detectors. The proposed model achieves an overall ${\mathrm{mAP}}_{50}$ of 0.902 and ${\mathrm{mAP}}_{50:95}$ of 0.447, showing notable improvements. Furthermore, TI-YOLO was successfully deployed on a low-cost embedded platform using the QAT method. Compared with YOLOv11n, TI-YOLO reduces FLOPs by 10.5% and parameters by 28.9%, while achieving an inference speed exceeding 50 FPS. These results indicate that TI-YOLO effectively balances detection accuracy and computational efficiency, meeting the real-time requirements of tracheal intubation assistance systems.

Overall, this work demonstrates the feasibility of applying lightweight deep learning models for real-time anatomical structure recognition in airway management. The proposed TI-YOLO framework provides a promising solution for intelligent assistance during tracheal intubation.

## Figures and Tables

**Table 1:** Detection performance comparison of different models on the laryngoscopic dataset.

Methods	Teeth	Tongue	Uvula	Epiglottis	CL-1	CL-2	CL-3	Carina	Esophagus	${\mathrm{mAP}}_{50}$	${\mathrm{mAP}}_{50:95}$
YOLOv5n	0.712±0.014	0.645±0.018	0.601±0.021	0.482±0.025	0.415±0.022	0.528±0.019	0.298±0.031	0.503±0.017	0.436±0.024	0.513±0.012	0.295±0.009
YOLOv8n	0.841±0.011	0.762±0.014	0.693±0.016	0.658±0.019	0.632±0.021	0.701±0.015	0.475±0.028	0.724±0.013	0.583±0.022	0.674±0.008	0.372±0.007
YOLOv10n	0.856±0.009	0.789±0.012	0.711±0.015	0.702±0.017	0.648±0.018	0.744±0.012	0.502±0.024	0.756±0.011	0.611±0.019	0.702±0.005	0.389±0.006
YOLOv11n	0.872±0.008	0.803±0.011	0.725±0.013	0.743±0.014	0.667±0.016	0.762±0.010	0.518±0.022	0.781±0.009	0.642±0.017	0.723±0.006	0.397±0.005
**TI-YOLO**	0.817±0.012	0.948±0.006	0.923±0.007	0.987±0.003	0.822±0.011	0.942±0.005	0.905±0.008	0.993±0.002	0.786±0.014	0.902±0.007	0.447±0.009

**Table 2:** Ablation study of TI-YOLO components.

BiFPN	DAT	SWF	Precision	Recall	${\mathrm{mAP}}_{50}$	${\mathrm{mAP}}_{50:95}$	mAPs
×	×	×	0.892	0.812	0.723	0.397	0.358
✔	×	×	0.904	0.825	0.741	0.414	0.381
×	✔	×	0.901	0.831	0.752	0.421	0.395
×	×	✔	0.898	0.838	0.758	0.426	0.404
✔	✔	×	0.912	0.843	0.781	0.438	0.423
✔	×	✔	0.918	0.848	0.821	0.443	0.432
×	✔	✔	0.916	0.851	0.845	0.445	0.438
✔	✔	✔	**0.929**	**0.855**	**0.902**	**0.447**	**0.445**

**Table 3:** Comparison of model complexity and deployment performance between YOLOv11n and TI-YOLO.

Model	QAT	GFLOPs	Parameters (M)	Model Size (MB)	FPS
YOLOv11n	×	6.5	2.6	5.4	35
TI-YOLO	×	5.8	1.8	4.6	38.6
**TI-YOLO**	✔	-	**1.8**	**2.9**	**>50**

## Data Availability

Data are contained within the article.
